# Periostin Safeguards EGFR‐Driven Genomic Instability and Sustains the Immune‐Suppressive Niche in Glioblastoma

**DOI:** 10.1155/humu/9501906

**Published:** 2026-05-03

**Authors:** Hongjun Liu, Shasha Tan, Jian Qi, Zhenjiang Du, Jinliang You, Sajjad Muhammad, Xiaoping Tang, Jianji Li

**Affiliations:** ^1^ Department of Neurosurgery, Affiliated Hospital of North Sichuan Medical College, Nanchong, Sichuan, China, hospital-nsmc.com.cn; ^2^ Department of Neurosurgery, Medical Faculty, University Hospital Düsseldorf, Heinrich-Heine-Universität, Düsseldorf, Germany, uni-duesseldorf.de; ^3^ Department of Rehabilitation Medicine, Affiliated Hospital of North Sichuan Medical College, Nanchong, Sichuan, China, hospital-nsmc.com.cn; ^4^ Department of Neurosurgery, The Fifth People′s Hospital of Nanchong, Nanchong, Sichuan, China; ^5^ Affiliated Hospital of Youjiang Medical University for Nationalities, Baise, Guangxi, China, gxyyfy.cn; ^6^ Key Laboratory of Research on Clinical Molecular Diagnosis for High Incidence Diseases in Western Guangxi of Guangxi Higher Education Institutions, Baise, Guangxi, China

**Keywords:** EGFR amplification, genomic instability, glioblastoma, immune-depleted microenvironment, periostin (POSTN), spatiotemporal evolution, tumor–myeloid crosstalk

## Abstract

Glioblastoma (GBM) heterogeneity limits the efficacy of EGFR‐targeted therapies. Here, we present a spatially stratified single‐cell atlas of IDH‐wildtype GBM to dissect the impact of EGFR amplification on tumor architecture. We demonstrate that EGFR amplification disrupts the spatial coupling between evolutionary state and anatomical location, resulting in premature acquisition of invasive phenotypes—a phenomenon we term “accelerated evolutionary velocity.” Unlike nonamplified tumors which maintain a strict “Core‐to‐Margin” developmental gradient, malignant cells in EGFR‐amplified tumors acquire invasive mesenchymal traits preemptively regardless of their spatial niche. This accelerated evolution parallels the Core behaving as a “genotoxic stress reservoir” characterized by elevated chromosomal instability (CIN) (*p* < 2.2 × 10^−16^). This genotoxic stress coincides with the emergence of a localized tumor–myeloid axis and an immune‐suppressive niche. Using the PriorityScore2 framework, we prioritized Periostin (POSTN) as a top‐tier clinically relevant candidate. In the high‐CIN environment of EGFR‐amplified GBM, in silico network perturbation suggested that POSTN may function as a candidate modulator of mitotic fidelity, potentially buffering against lethal genomic instability while sustaining rapid clonal evolution. Validated across multicenter cohorts, POSTN showed robust upregulation, strong diagnostic performance (AUC = 0.961), and significant prognostic relevance, emerging as a potential therapeutic vulnerability linking accelerated evolution with immune privilege in the GBM ecosystem.

## 1. Introduction

Somatic structural variation (SV) and chromosomal instability (CIN) constitute the bedrock of tumor evolution, dictating cellular fitness, phenotypic plasticity, and the emergence of therapeutic resistance [1]. While historical analyses have largely focused on the cataloging of single‐nucleotide variants, contemporary cancer genomics has shifted toward elucidating the functional consequences of large‐scale somatic copy‐number alterations (SCNAs). In solid tumors, particularly those with high mutational burdens, SCNAs are not merely passenger events but active drivers that reshape the transcriptome, creating permissive landscapes for clonal diversification [2]. Recent pan‐cancer analyses highlight a critical, yet underexplored paradigm: how intrinsic genomic instability propagates into extrinsic microenvironmental remodeling. Specifically, high levels of aneuploidy and CIN have been increasingly linked to the formation of “immune‐depleted microenvironment” and myeloid polarization, suggesting that SV acts as a cell‐autonomous signal that orchestrates immune evasion. Consistent with this paradigm, cohort‐scale transcriptomic analyses of glioblastoma (GBM) have shown that tumor‐intrinsic signaling nodes can be statistically associated with inferred immune cell infiltration patterns, supporting the interpretability of transcriptome‐derived immune deconvolution as an orthogonal line of evidence for tumor–immune coupling [3].

GBM, the most lethal primary malignancy of the central nervous system, represents the archetype of a disease driven by structural genomic chaos [4]. The genomic landscape of IDH‐wildtype GBM is dominated by complex SCNAs, with Epidermal Growth Factor Receptor (EGFR) amplification serving as a hallmark event occurring in approximately 40% of cases [5]. Although EGFR amplification is routinely utilized for molecular stratification, its role extends far beyond a static diagnostic marker. It is a highly dynamic driver, often residing on extrachromosomal DNA (ecDNA) or undergoing heterogeneous retention, which underpins the dramatic intratumoral heterogeneity observed in patients [6]. However, a critical disconnect persists in our understanding: Despite the prevalence of EGFR amplification, targeted therapies have consistently failed. This failure is likely attributable to the spatial uncoupling of genotype and phenotype, where distinct anatomical niches (e.g., the hypoxic core vs. the invasive Margin) impose divergent evolutionary selection pressures that modulate the impact of the driver mutation.

Current mutational studies often treat tumors as “bulk” mixtures or dissociate cells from their spatial context, thereby obscuring the localized interplay between genetic drivers and environmental stress [7, 8]. A significant gap exists in quantifying how driver copy number variations (CNVs), such as EGFR amplification, differentially modulate evolutionary velocity and genomic instability across the tumor′s architectural zones [9]. Does the Tumor Core serve merely as a necrotic endpoint, or does it function as a mutagenic reservoir that actively generates subclonal diversity through sustained genotoxic stress? Furthermore, how does this localized genomic stress translate into the immunosuppressive myeloid barriers that characterize GBM? Addressing these questions requires a move beyond static mutation calling toward spatiotemporal integration, where CNV burden, transcriptomic trajectories, and immune interactions are mapped as coupled systems [10]. Emerging single‐cell spatial technologies and computational inference of CNV states now provide the resolution necessary to reconstruct these hidden evolutionary architectures [11, 12].

Here, we leverage a spatially stratified single‐cell transcriptomic atlas of IDH‐wildtype GBM to deconstruct the evolutionary logic of EGFR amplification. By integrating trajectory inference with inferred single‐cell CNV profiling, we demonstrate that EGFR amplification acts as a structural catalyst that reorganizes tumor evolution. We quantify a phenomenon of “Evolutionary Acceleration,” wherein EGFR‐amplified cells in the Tumor Core preemptively acquire invasive phenotypes driven by intense, niche‐specific CIN. Furthermore, we establish a mechanistic link between this genomic instability and the establishment of an innate immune‐depleted microenvironment, mediated by a spatially restricted “tumor–myeloid axis.” Finally, employing a multicriterion prioritization strategy (PriorityScore2) and in silico network perturbation, we prioritize Periostin (POSTN) as a candidate molecular brake governing this eco‐evolutionary process. Collectively, this work advances the field of human mutation by providing a quantitative framework that links recurrent SVs to spatially resolved phenotypic kinetics and actionable microenvironmental vulnerabilities.

## 2. Materials and Methods

### 2.1. Study Design and Analytical Workflow

We constructed a spatially stratified single‐cell atlas of IDH‐wildtype GBM to dissect how EGFR amplification reshapes tumor evolution and immune ecology (Figure S1). Transcriptomes from EGFR‐amplified and nonamplified tumors were sampled across the Tumor Core and the invasive Margin. Seurat‐based preprocessing enabled cell‐type annotation and functional module scoring. Evolutionary trajectories were reconstructed using Monocle 3 pseudotime analysis, revealing accelerated malignant state progression in EGFR‐amplified niches. Copy‐number alterations and CIN were inferred with inferCNV, identifying the Core as a genotoxic stress reservoir. Spatial tumor–immune coupling was quantified through polarized myeloid programs, defining a localized tumor–myeloid axis. PriorityScore2 and virtual perturbation nominated POSTN as a candidate vulnerability sustaining immune privilege and CIN tolerance.

### 2.2. Spatially Stratified Cohort Design and Cell Type Annotation

We curated a high‐resolution single‐cell transcriptomic atlas from six IDH‐wildtype GBM patients (GSE286419) [13], employing a strict 2 × 2 factorial design stratified by EGFR amplification status (EGFR_amp vs. non_EGFR_amp) and intratumoral spatial localization (Tumor Core vs. Margin) (Table S1). To ensure data fidelity, raw feature matrices underwent rigorous quality control: Cells with mitochondrial gene content > 20% and < 200 detected features were excluded to remove apoptotic debris. Data normalization was performed using SCTransform in Seurat (V5) [14], regressing out cell cycle scores and mitochondrial percentage. Batch effects were corrected using the Harmony algorithm (theta = 2, max.iter.cluster = 20), followed by uniform manifold approximation and projection (UMAP). Cell types were annotated based on canonical markers (e.g., EGFR for tumor cells, CD3D for T cells, and C1QA for macrophages). Functional states, such as “Cytotoxicity Score” and “Immunosuppressive Score,” were quantified using the AddModuleScore function based on curated gene signatures derived from Molecular Signatures Database (MSigDB) [15]. Tumor Purity was mathematically defined as the fraction of cells harboring malignant copy number events within each spatial niche.

### 2.3. Trajectory Inference and Quantification of Evolutionary Acceleration

To reconstruct the continuous transcriptomic landscape, we utilized Monocle 3 with a reverse graph embedding algorithm [16]. The trajectory root was biologically defined as the “NPC‐like” population enriched in the Tumor Core. We introduced a quantitative framework to measure Evolutionary Acceleration: (1) Kinetic Modeling: We applied Local Polynomial Regression (Loess) to model the slope of mesenchymal (MES) score acquisition as a function of pseudotime; (2) Pseudotime Shift Analysis: We calculated the divergence in cell density distribution between EGFR groups using the Jensen–Shannon divergence metric. The “Endpoint‐program” was defined as cells falling within the top quartile (75th percentile) of global MES scores.

### 2.4. Inferred Genomic Instability and Spatial Phylogenetics

Large‐scale CNVs were inferred using infercnv (V1.24.0) [17]. Oligodendrocytes and immune cells served as the diploid reference baseline. To preserve subclonal heterogeneity, the Hidden Markov Model (HMM) was disabled. We quantified CIN using a custom “CNV Score,” calculated as the mean squared deviation of expression from the diploid baseline: CIN = (Observed − Expected)2. Scores were normalized to a [−1, 1] scale. To capture spatial genetic polarization, we compared the variance of chromosome arm‐level expression profiles between Core and Margin regions using Levene′s test for equality of variances. For spatial genetic polarization analysis using Levene′s test, the statistical unit was defined at the region‐summarized cell level, comparing the variance of chromosome arm‐level expression scores across all individual malignant cells within the Core versus those in the Margin.

### 2.5. Tumor–Myeloid Coupling Framework and Priority Scoring

We developed a sample‐level tumor–myeloid interaction framework to prioritize candidate mediators of malignant evolution. A data‐driven myeloid gene module was first identified by correlating myeloid gene expression with the malignant endpoint program across samples, and the mean expression of the selected genes was used to calculate MyeloidAutoScore for each sample. Candidate genes were evaluated within a focused set of pseudotime late‐up genes that were detectable in malignant cells and, when indicated, restricted to curated ligand annotations. For each candidate, tumor–myeloid coupling strength (coup_rho) was defined as the Spearman correlation between its mean expression in late‐stage malignant cells and the matched sample‐level MyeloidAutoScore across samples.

We then defined PriorityScore2 as 0.40 × z_coup + 0.35 × z_late + 0.25 × z_egfr, where z_coup represents tumor–myeloid coupling, z_late represents late‐state enrichment, and z_egfr represents EGFR‐amplified specificity. Only positively coupled candidates (coup_rho > 0) were retained in the final hard‐gated ranking, and alternative weight settings were tested to assess robustness. This prioritization was designed as a focused candidate‐screening framework rather than a genome‐wide discovery model.

### 2.6. Virtual Perturbation

To simulate the functional impact of candidate targets, we employed scTenifoldKnk (V1.0.3) [18]. A single‐cell Gene Regulatory Network (GRN) was constructed using the PC‐net algorithm. A “virtual knockout” (vKO) of POSTN was performed by zeroing out its outgoing weights in the adjacency matrix. The perturbation effect was quantified by identifying genes with significant topological shifts (Manifold Alignment Distance) between the wild‐type and vKO networks. Functional consequences were adjudicated using Gene Set Enrichment Analysis (GSEA) on the rank‐ordered list of perturbed genes (|Z| > 1.96).

### 2.7. Statistical Analysis

Statistical analyses were performed according to data types. Single‐cell level comparisons (e.g., differential expression and pseudotime) used the Wilcoxon rank‐sum test. Sample‐level spatial comparisons used paired or two‐sample Student′s *t*‐tests, as appropriate. Linear regression was applied to evaluate the tumor–myeloid coupling. For clinical validation, diagnostic performance was assessed by receiver operating characteristic (ROC) curves (AUC), and survival was analyzed using Kaplan–Meier curves with the log‐rank test. A two‐sided *p* value < 0.05 was considered statistically significant.

## 3. Results

### 3.1. EGFR Amplification Orchestrates an Immune‐Desertified Spatial Architecture

**Figure 1:**
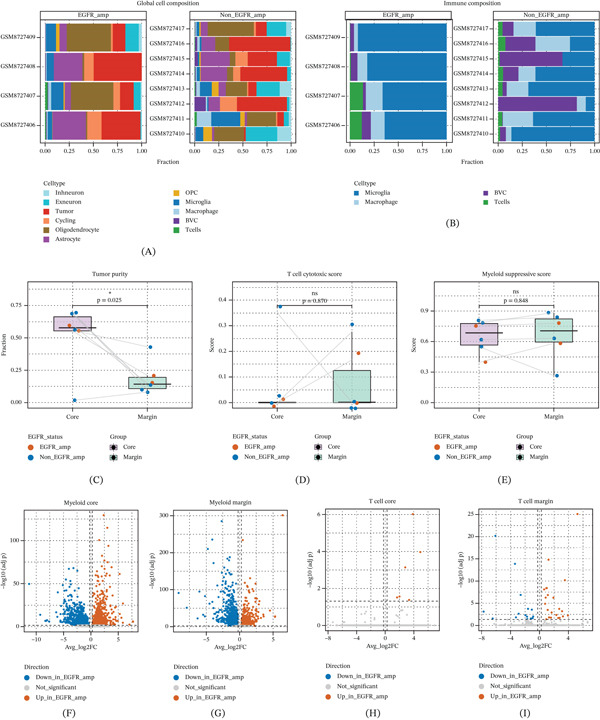
EGFR amplification drives spatial immune desertification and functional suppression. (A) Global cell type composition across EGFR states. (B) Immune subset proportions revealing significant depletion in EGFR‐amplified tumors. (C) Tumor purity analysis validating spatial sampling and invasive potential. (D–E) Functional scoring revealing (D) globally low T‐cell cytotoxicity and (E) broadly established myeloid immunosuppressive signatures, both of which are independent of spatial niche. (F–I) Volcano plots of differential expression in (F–G) myeloid and (H–I) T cells across spatial niches.  ^∗^ *p* < 0.05, ns: not significant.

To systematically characterize the spatial, evolutionary, and immune consequences of EGFR amplification in IDH‐wildtype GBM, we established an integrated analytical framework incorporating single‐cell atlas profiling, trajectory analysis, CIN/CNV assessment, tumor–myeloid coupling, candidate prioritization, and multicohort validation. Global decomposition of the single‐cell atlas revealed marked remodeling of the GBM ecosystem according to EGFR amplification status (Figure S2A). Cell‐type annotations were supported by canonical marker expression patterns, including EGFR in tumor cells, CD3D/TRAC in T cells, C1QA/CD163 in myeloid cells, and lineage markers for oligodendrocytes, astrocytes, and neuronal populations (Figure S2D). At the whole‐ecosystem level, sample‐wise cell composition analysis showed that both EGFR‐amplified (EGFR_amp) and nonamplified tumors were dominated by malignant and lineage‐associated nonmalignant brain cell populations, whereas T‐cell abundance remained uniformly low in both groups (Figure 1A; Figure S2B,C). When the immune compartment was examined separately, EGFR_amp tumors showed reduced innate immune infiltration, most notably a lower proportion of macrophages and a modest decrease in microglia compared with non_EGFR_amp tumors, whereas T‐cell abundance remained minimal in both groups (Figure 1B; Figure S2B,C). Consistent with these compositional differences, tumor purity was significantly higher in the Core than in the Margin across patients (*p* = 0.025; Figure 1C), with EGFR_amp samples showing particularly limited nonmalignant representation in the Core. Functional scoring further demonstrated uniformly low T‐cell cytotoxic activity (*p* = 0.870; Figure 1D) and broadly high myeloid immunosuppressive signatures (*p* = 0.848; Figure 1E**)** without significant spatial discrepancies, likely reflecting a generalized recruitment of innate immune cells at the tumor–normal interface. Differential expression analysis of myeloid and T‐cell compartments further identified niche‐ and EGFR‐associated transcriptional differences in both immune lineages (Figure 1F–I). Together, these findings suggest that EGFR amplification is associated with a more tumor‐enriched ecosystem, reduced innate immune infiltration, and spatially structured differences in baseline immune functional states. Importantly, although the relative proportion of myeloid cells is reduced in EGFR_amp tumors, their immunosuppressive transcriptional programs are spatially reinforced within the Core, revealing a critical divergence between immune cell quantity and functional polarization.

### 3.2. EGFR Amplification Catalyzes an Accelerated Evolutionary Trajectory

**Figure 2:**
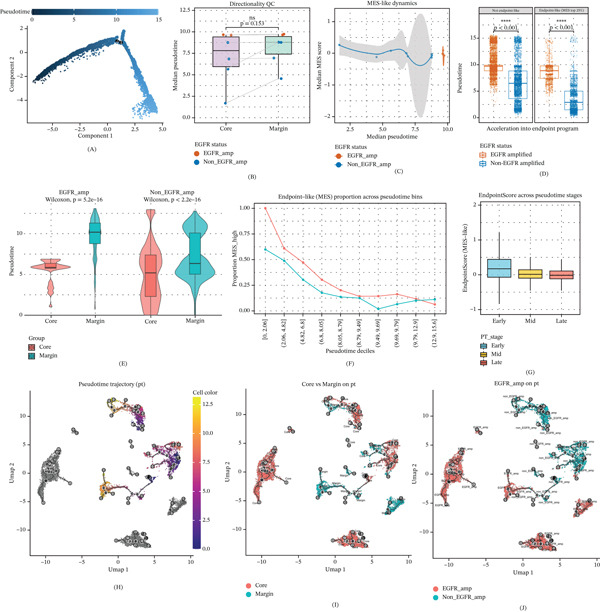
EGFR amplification accelerates spatiotemporal evolutionary dynamics. (A) Monocle pseudotime trajectory of malignant cells. (B) Spatial distribution of pseudotime across niches, demonstrating that EGFR amplification effectively flattens the spatial evolutionary gradient. (C) Loess regression modeling the accelerated rate of mesenchymal acquisition. (D–F) Quantification of preemptive “endpoint‐like” states, with panel D confirming a significantly higher pseudotime distribution in EGFR‐amplified cells via cell‐level Wilcoxon rank‐sum tests. (G) Distribution of mesenchymal scores across evolutionary phases. (H–J) Trajectory mappings of (H) pseudotime, (I) spatial location, and (J) EGFR status confirming evolutionary uncoupling driven by EGFR amplification.  ^∗∗∗∗^ *p* < 0.0001, ns: not significant.

Pseudotime trajectory reconstruction resolved the developmental hierarchy of GBM, mapping a continuous lineage from progenitor‐like states in the Tumor Core to mesenchymal (MES‐like) states at the invasive Margin (Figure 2A; Figure S3A). This progression was characterized by the gradual decay of NPC/OPC‐like signatures and the monotonic acquisition of invasive mesenchymal traits (Figure 2G; Figure S3C–F), consistent with the established NPC‐AC‐MES‐like differentiation hierarchy in GBM [19]. Projecting anatomical coordinates onto this axis revealed a strict topological alignment in nonamplified tumors, where evolutionary maturation spatially mirrored the “Core‐to‐Margin” invasion axis **(**Figure 2E,I**)**. In contrast, EGFR amplification dismantled this spatial constraint, enforcing an “Accelerated Evolutionary Velocity” across the ecosystem. Consequently, the global pseudotime gradient between the Core and Margin was effectively flattened (*p* = 0.153; Figure 2B). Malignant cells in EGFR‐amplified tumors occupied late‐stage pseudotime bins regardless of their spatial niche **(**Figure 2E**)**, maintaining a significantly higher pseudotime distribution and frequency of endpoint‐like states (cell‐level Wilcoxon rank‐sum test, *p* < 0.001; Figure 2D,F) and a steeper slope of mesenchymal acquisition compared to nonamplified controls (Figure 2C,J). Mechanistically, this acceleration was driven by the premature activation of “late‐up” matrix‐remodeling programs (e.g., POSTN) (Figure S3B). Collectively, these data identify EGFR amplification as an evolutionary catalyst that lowers the activation energy for phenotypic plasticity, enabling the preemptive acquisition of invasive properties prior to physical dissemination.

### 3.3. The Tumor Core Acts as a Reservoir of Genomic Instability and Clonal Stress

**Figure 3:**
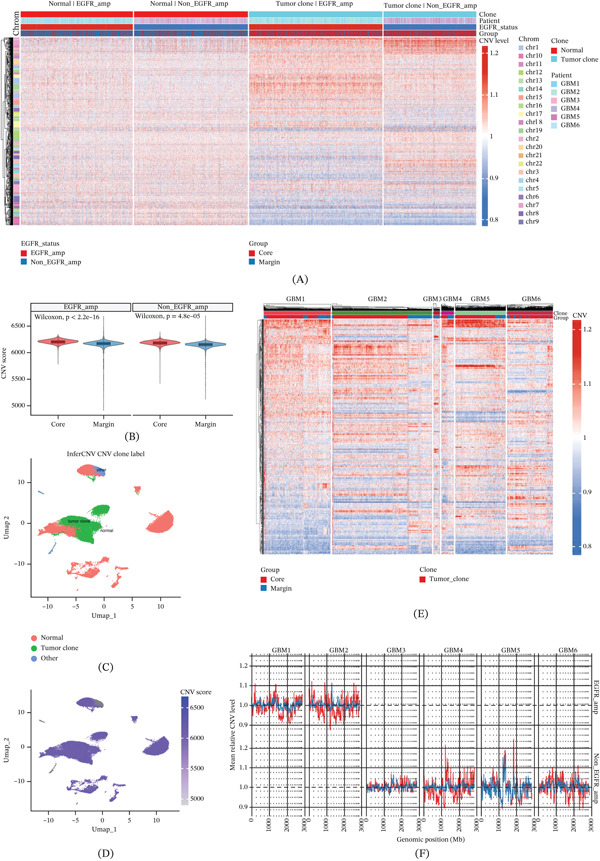
Spatial genomic heterogeneity and subclonal CNV architecture. (A) Genome‐wide inferCNV heat map showing global chromosomal alteration patterns across normal and tumor‐clone populations, stratified by EGFR status and spatial group. (B) Violin plots comparing CNV scores between Core and Margin malignant cells in EGFR_amp and non_EGFR_amp tumors. (C) UMAP visualization of inferCNV clone labels, distinguishing normal, tumor‐clone, and other cell populations. (D) UMAP projection of CNV scores across the integrated single‐cell atlas. (E) Patient‐level CNV heatmaps showing recurrent spatially stratified subclonal CNV patterns across individual tumors. (F) Chromosome‐scale mean CNV profiles comparing genomic deviation amplitudes between spatial groups across patients.

Having established that EGFR amplification is associated with accelerated malignant state progression, we next asked whether this pattern was accompanied by increased genomic instability. We therefore performed genome‐wide CNV inference to map the spatial architecture of chromosomal alterations across malignant cells. Genome‐wide copy number profiling distinguished normal and tumor‐clone populations and revealed broad chromosomal alteration patterns across the malignant ecosystem (Figure 3A,C,D). Quantitative CNV scoring showed that malignant cells in the Tumor Core harbored significantly higher CNV burden than those in the Margin in both EGFR_amp and non_EGFR_amp tumors (*p* < 2.2 × 10^−16^ and *p* = 4.8 × 10^−5^) (Figure 3B). Patient‐level CNV heat maps further showed that this spatial difference was not restricted to a single case but recurred across multiple samples, with stronger subclonal deviation patterns generally observed in Core‐derived tumor cells relative to Margin‐derived counterparts (Figure 3E). Consistently, chromosome‐scale mean profiles also supported greater deviation amplitude in Core‐associated malignant populations, particularly in EGFR‐amplified tumors (Figure 3F). Together, these data indicate that the Tumor Core represents a spatial niche with elevated CIN and subclonal stress, rather than a genetically static tumor compartment.

### 3.4. The Genetically Unstable Core Orchestrates a Localized Immune Shield

The concentration of genomic instability in the Core raised a pivotal question: Does this genotoxic stress actively shape a specialized immune microenvironment? While global transcriptional signatures indicated high baseline myeloid suppression at the invasive Margin, we hypothesized that the intensified evolutionary and genetic activity within the Core might orchestrate a distinct, niche‐specific immune program.

**Figure 4:**
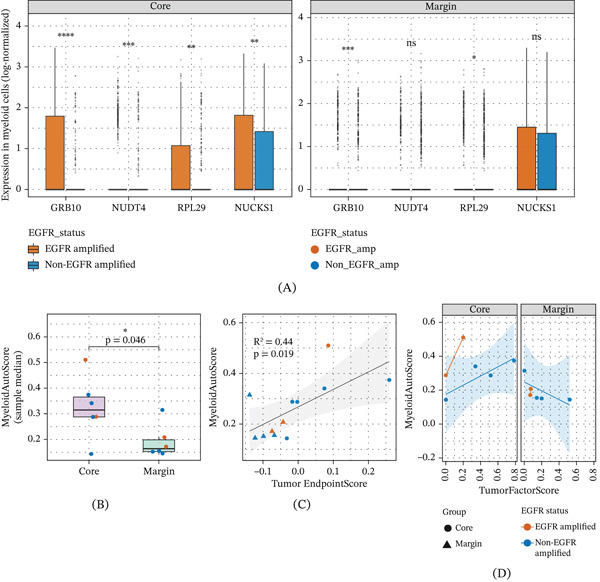
The Tumor Core orchestrates myeloid polarization and evolutionary coupling. (A) Expression levels of spatially polarized myeloid genes (e.g., GRB10) evaluated at the single‐cell level, peaking significantly within the Tumor Core of EGFR‐amplified samples. (B) MyeloidAutoScore demonstrating significantly elevated evolution‐linked immunosuppression in the Core compared to the Margin across patients. (C) Global linear regression revealing a significant positive coupling between the tumor evolutionary endpoint and myeloid suppression. (D) Stratified trendline visualization confirming the strongest tumor–myeloid coupling within the EGFR‐amplified Core niche, defining a distinct “high‐evolution/high‐suppression” niche.  ^∗^ *p* < 0.05,  ^∗∗^ *p* < 0.01,  ^∗∗∗^ *p* < 0.001,  ^∗∗∗∗^ *p* < 0.0001, ns: not significant.

To address this, we systematically profiled myeloid gene modules across spatial niches. Unbiased correlation screening identified a spatially polarized module of myeloid genes (e.g., GRB10, NUCKS1) whose expression mirrored the trajectory of tumor evolution—peaking specifically in the Tumor Core of EGFR‐amplified tumors (cell‐level Wilcoxon test, *p* < 0.05 to *p* < 0.0001; Figure 4A) and decaying toward the Margin. Aggregation of these features into a sample‐level MyeloidAutoScore (representing evolution‐linked suppression) revealed that this specialized immunosuppression was significantly elevated in the Core compared to the Margin across patients (*t*‐test, *p* = 0.046; Figure 4B), creating a localized “immune shield” around the evolutionary hub.

Regression analysis further uncovered a significant global linear coupling (*R* ^2^ = 0.44, *p* = 0.019) between the malignant “Endpoint‐program” and this myeloid axis (Figure 4C). While spatial sample size limitations precluded formal subgroup regression statistics, trendline analysis supported that this tumor–myeloid coupling was most pronounced within the Tumor Core, defining a distinct “high‐evolution/high‐suppression” niche (Figure 4D). Crucially, this interaction exhibited strict spatial specificity: The regression slope connecting tumor factors and myeloid suppression was visibly steeper in the EGFR‐amplified Core compared to the Margin. This indicates that while general immune exclusion is prominent at the Margin, the specific reciprocal crosstalk driven by genomic instability is topologically confined to the Core. These data establish a “tumor–myeloid axis” where the genetically unstable Core acts as a signaling hub, orchestrating a protective niche that likely facilitates subsequent invasion.

### 3.5. PriorityScore2 Prioritizes POSTN as a Top‐Tier and Clinically Accessible Target

**Figure 5:**
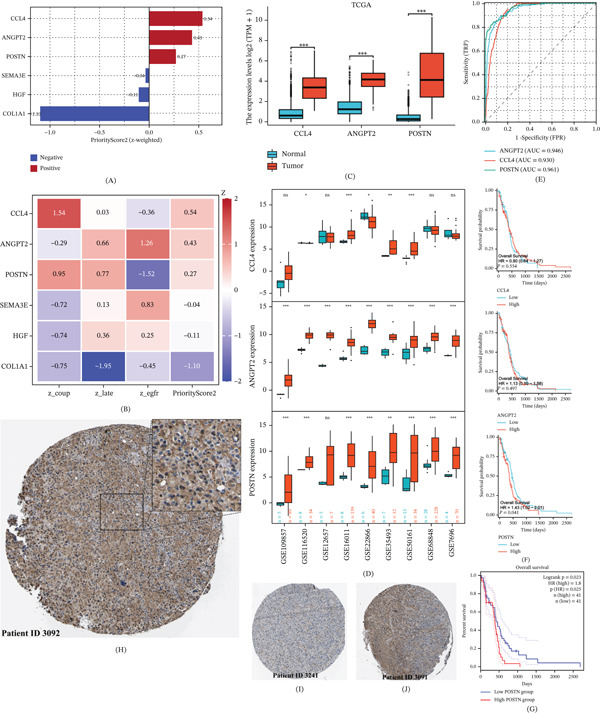
Prioritization of POSTN as a top‐tier clinically relevant candidate. (A–B) PriorityScore2 ranking and component analysis identifying CCL4, ANGPT2, and POSTN as top‐tier candidates, with POSTN supported by strong tumor–myeloid coupling and late‐state enrichment. (C–D) Validation of POSTN upregulation in TCGA and nine independent GEO cohorts. (E) Receiver operating characteristic (ROC) curve evaluating the diagnostic performance of POSTN in the TCGA glioblastoma cohort (AUC = 0.961). (F–G) Survival analyses evaluating the prognostic relevance of POSTN in the (F) TCGA cohort and the (G) GEPIA2 platform. (H–J) Representative immunohistochemistry (IHC) images showing POSTN protein expression in high‐grade malignant glioma tissues. Images were obtained from the Human Protein Atlas (HPA) database using antibody HPA012306 (Panel H: Patient ID 3092, male, age 48, with a magnified inset; Panel I: Patient ID 3241, female, age 58; Panel J: Patient ID 3091, male, age 71).

To translate these findings into clinically actionable targets, we applied the multidimensional PriorityScore2 framework, integrating tumor–myeloid coupling, late‐state activation, and EGFR‐amplified specificity. This focused ranking identified CCL4, ANGPT2, and POSTN as the top‐tier candidates (Figure 5A). Within this framework, POSTN ranked third, with strong support from tumor–myeloid coupling (z_coup = 0.95) and late‐state enrichment (z_late = 0.77), whereas weaker EGFR‐amplified specificity reduced its composite score relative to CCL4 and ANGPT2 (Figure 5B). Notably, sensitivity analyses across alternative weighting schemes showed that POSTN consistently remained within the top tier, ranking second or third in all tested models (Figure 4).

We then evaluated these candidates using independent clinical and validation datasets. Although CCL4 and ANGPT2 showed promising diagnostic signals, neither was significantly associated with patient survival (*p* > 0.05) (Figure 5F), and ANGPT2 exhibited less stable expression across selected validation cohorts (Figure 5D). By contrast, POSTN showed robust upregulation across TCGA and nine independent GEO cohorts, superior diagnostic accuracy (AUC = 0.961) (Figure 5C–E), significant association with poor overall survival (HR = 1.43, *p* = 0.041) (Figure 5F), and independent support from GEPIA2 (HR = 1.8, *p* = 0.023) (Figure 5G). IHC further demonstrated dense POSTN accumulation in the extracellular matrix at the invasive front, underscoring its accessibility and its central role in the tumor–myeloid shield (Figure 5H–J). Together, these data support POSTN as the most clinically relevant candidate emerging from the prioritization framework.

### 3.6. In Silico Perturbation Suggests POSTN as a Potential Stabilizer of Mitotic and Remodeling Programs

**Figure 6:**
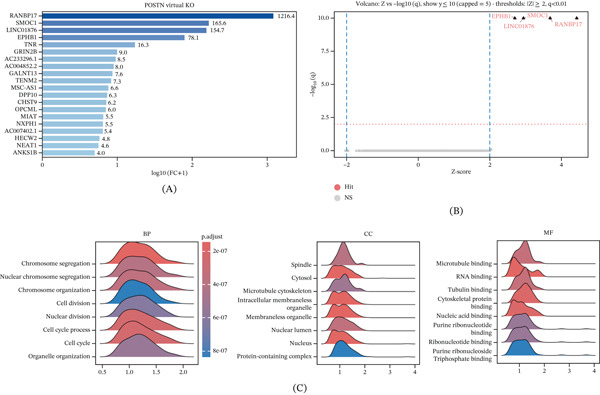
Virtual knockout reveals POSTN as a stabilizer of the mitotic network. (A) Top upregulated genes (e.g., *RANBP17*, *SMOC1*) following *POSTN* virtual knockout (vKO). (B) Volcano plot showing unilateral upregulation of invasion programs upon vKO. (C) GSEA highlighting the systemic reactivation of microtubule, spindle, and chromosome segregation pathways in the absence of *POSTN*.

To dissect the regulatory logic of POSTN, we performed in silico vKO analysis on malignant cells at the evolutionary terminus. This perturbation revealed a substantial, unilateral repatterning of the transcriptome. Differential regulation analysis showed that POSTN deficiency triggered the explosive upregulation of a specific gene module, with RANBP17 (FC = 1216.4), SMOC1 (FC = 165.6), and LINC01876 ranking as the top effectors, followed by key mediators such as EPHB1 and TNR (Figure 6A). This architecture was structurally confirmed by the volcano plot, where significant perturbations were exclusively confined to the upregulation quadrant, identifying POSTN as an essential molecular brake (Figure 6B). GSEA further highlighted that this unleashed program reactivates the “Microtubule–Spindle–Chromosome” axis, with significant enrichment in chromosome segregation and spindle organization (Figure 6C). These data suggest that POSTN normally imposes a stabilizing constraint on the mitotic machinery, and its loss precipitates a transcriptomic state associated with heightened genomic instability and structural remodeling.

## 4. Discussion

EGFR amplification in IDH‐wildtype GBM is often framed as a proliferative driver, yet its clinical tractability has remained disappointing [20]. Our spatially stratified single‐cell analyses suggest that EGFR amplification may function as an eco‐evolutionary catalyst, reshaping how malignant transcriptional states are distributed across anatomical niches [9]. Recent integrative spatial studies propose that gliomas follow reproducible organizational principles—local environments enriched for dominant tumor states and recurrent spatial pairings—rather than a strictly linear “Core‐to‐Margin” continuum [21]. In this context, our observation of genotype–phenotype uncoupling in EGFR‐amplified tumors suggests that amplification can relax spatial constraints on state acquisition, allowing invasion‐associated programs to emerge earlier within the Core than would be expected from anatomy alone.

A plausible mechanistic explanation for this “accelerated evolutionary velocity” is supported by the biology of ecDNA and high‐level oncogene amplification. ecDNA can provide a non‐Mendelian substrate that enables rapid copy‐number fluctuation, transcriptional bursts, and heterogeneous retention across space—properties that could decouple anatomical location from evolutionary state [22–24]. Importantly, recent work has begun to define actionable vulnerabilities of ecDNA‐positive cancers, including strategies that exploit transcription–replication conflict, conceptually aligning with a model in which stress‐amplified evolution is concentrated within EGFR‐driven niches [25]. Together, these observations suggest a rationale for why EGFR‐directed therapies can yield limited durability in GBM: The target may be embedded within a dynamic amplification architecture that promotes phenotypic plasticity and rapid evolutionary exploration, constraining sustained suppression [26, 27].

Our data further highlight a spatial paradox: The Tumor Core, often interpreted clinically as necrotic or terminal, may also act as an evolutionary engine in which CIN and subclonal stress are concentrated [28, 29]. This view is consistent with broader spatial omics literature emphasizing that hypoxic, stressed niches—potentially driven by intense local oxidative stress—are not merely passive endpoints but can act as evolutionary engines that seed poor‐prognosis architectures [30]. Notably, CIN has dual immunological consequences: While cytosolic DNA signaling can trigger innate immune activation in some contexts, the net effect in GBM frequently favors immune dysfunction and myeloid‐dominant suppression [31]. In our cohort, the Core′s elevated genomic stress coincided with a localized strengthening of a tumor–myeloid coupling program, suggesting that genotoxic pressure is not only tolerated but may be leveraged to sculpt a protective microenvironment.

This places the immune compartment into the causal loop. GBM is increasingly recognized as a myeloid‐driven immunosuppressive disease in which macrophages/microglia and their polarization states dominate local immune tone, whereas T‐cell cytotoxicity is often blunted [32, 33]. Our finding that immunosuppressive myeloid programs couple most strongly to late‐evolution malignant states in the Core provides a spatially resolved instantiation of this principle. Public cohort‐scale analyses also support the utility of transcriptome‐derived immune infiltration inference to validate tumor–immune statistical coupling in GBM, offering an orthogonal layer to spatial single‐cell observations [34]. A key implication is that immune privilege in GBM may be better conceptualized as a niche‐confined adaptive state—reinforced where evolutionary stress is maximal—rather than a uniform tumor‐wide attribute [35]. Furthermore, while our transcriptomic inference highlights myeloid polarization, downstream effectors likely involve complex metabolic adaptations; it is plausible that EGFR‐driven signaling in the Core orchestrates metabolic rewiring of these recruited myeloid cells, such as a shift toward glycolysis, which remains an important direction for future experimental validation.

Our findings reveal POSTN as a paradoxical “molecular brake” in the GBM ecosystem. While EGFR amplification catalyzes accelerated evolutionary velocity, the resulting CIN may increase the risk of deleterious or even lethal genomic imbalance when instability exceeds a tolerable window [36]. We propose that POSTN functions not as a tumor suppressor but as a mitotic rheostat that helps maintain genomic integrity within a viable threshold. Mechanistically, POSTN may influence mitotic fidelity via integrin‐linked adhesion signaling that couples cytoskeletal organization with spindle positioning during mitosis, thereby limiting the transition from adaptive instability to catastrophic collapse and enabling high‐stress core cells to exploit subclonal diversity without crossing a lethal boundary [37]. Crucially, in this context, this “brake” does not slow down tumor evolution; rather, by preventing catastrophic collapse under extreme genomic stress, it paradoxically enables the sustained and accelerated evolution of the EGFR‐amplified Core.

Within this framework, POSTN emerges as a particularly compelling hub because it can plausibly unify extracellular matrix remodeling, immune niche conditioning, and stress tolerance. POSTN is a secreted extracellular matrix glycoprotein (a matricellular protein) that engages integrin‐centered signaling to modulate cell adhesion, migration, and stromal–immune interactions [38]. Classic mechanistic work demonstrated that glioma stem‐like cells can secrete POSTN to recruit and educate tumor‐associated macrophages (TAMs), enriching an immunosuppressive perivascular niche that supports tumor growth [39]. Our data extend this niche concept by positioning POSTN as a molecular “brake” that may support mitotic fidelity under high‐CIN conditions, thereby potentially limiting catastrophic genomic collapse while sustaining the evolutionary throughput of the Core. In other words, POSTN may help maintain the viability of an otherwise genotoxically precarious ecosystem, enabling the tumor to benefit from CIN‐driven diversification without crossing the threshold into lethal instability. Importantly, we do not interpret PriorityScore2 as a standalone proof that POSTN is universally the highest‐ranked ligand under all possible scoring schemes. Rather, the framework was designed to identify top‐tier candidates within a biologically constrained set, after which convergent diagnostic, prognostic, and histopathological evidence supported POSTN as the most clinically relevant target.

Furthermore, the extracellular and secreted nature of POSTN presents unique translational opportunities and challenges [39]. While the blood–brain barrier (BBB) typically restricts large‐molecule penetrance, the blood–tumor barrier in GBM is spatially heterogeneous, and relatively angiogenic tumor‐core regions may provide only a partial window for targeted delivery rather than uniformly permitting drug access [40]. Notably, POSTN has been widely reported as a stromal matricellular protein enriched in cancer‐associated fibroblasts (CAFs) and implicated in tumor progression and therapy resistance across multiple solid tumors [41]. In the specialized microenvironment of GBM, this functional niche may be at least partially occupied by perivascular fibroblasts and related mural‐associated stromal populations [42]. Our findings therefore suggest that POSTN+ cells in the GBM microenvironment may function analogously to POSTN+ CAFs, contributing to extracellular matrix deposition, spatial immune exclusion, and myeloid‐skewed immunosuppression. Consequently, targeting the POSTN axis may help remodel stromal barriers and provides a rationale for testing combination strategies with immune checkpoint blockade. Given the intrinsically immune‐cold nature of GBM and the limited efficacy of PD‐1 blockade alone [43], POSTN‐targeted interventions may represent a plausible strategy to improve T‐cell access and sensitize selected tumors to immunotherapy, although this hypothesis still requires direct validation in GBM‐specific preclinical models.

Therapeutically, this duality suggests an attractive hypothesis: Targeting POSTN (or its immediate signaling dependencies) could simultaneously weaken the immunosuppressive myeloid shield and destabilize the mitotic tolerance program that permits sustained evolution in EGFR‐amplified niches [44]. Such a two‐axis disruption is conceptually distinct from classical EGFR inhibition, because it attacks the ecological supports that make EGFR‐driven amplification and evolution clinically resilient. More broadly, our results argue that effective intervention in EGFR‐amplified GBM may require a “niche‐aware” design—one that integrates genomic instability, amplification architecture, and myeloid ecology as coupled systems rather than separable hallmarks [45].

Several limitations should be considered. First, pseudotime‐based inference of evolutionary acceleration, while informative, will benefit from orthogonal validation in lineage‐tracing systems and patient‐derived models that preserve spatial constraints [46]. Second, the molecular route by which POSTN constrains mitotic instability under high CIN remains to be experimentally mapped at high resolution, including potential integrin/FAK‐dependent coupling to spindle or chromosome segregation machinery [47]. Third, the myeloid programs captured here may contain distinct microglial versus monocyte‐derived macrophage subcircuits with different therapeutic tractability; resolving these subcomponents will be important for translating POSTN‐centered strategies into combinatorial regimens [48, 49]. Despite these limitations, our study provides a quantitative framework linking EGFR amplification to spatially accelerated evolution, Core‐confined CIN stress, and a locally reinforced tumor–myeloid immune shield, highlighting POSTN as a strategic vulnerability in the GBM ecosystem.

## 5. Conclusions

In conclusion, our study provides new insights into how EGFR amplification accelerates the evolutionary process in GBM through genomic instability and immune microenvironment remodeling. We found that EGFR amplification not only accelerates tumor cell evolution but also creates a synergistic coupling between accelerated evolution and immune privilege, which supports tumor immune evasion. POSTN, as a key molecule in this process, may serve as a target for therapy, disrupting both the mitotic stability and the immune shield of the tumor, providing a potential strategy for GBM treatment.

## Author Contributions

**Hongjun Liu:** formal analysis, software, conceptualization, project administration, writing – original draft. **Shasha Tan:** conceptualization, methodology, formal analysis, visualization. **Jian Qi:** methodology, validation. **Zhenjiang Du:** software, data curation. **Jinliang You:** methodology, data curation. **Sajjad Muhammad:** methodology, conceptualization. **Xiaoping Tang:** supervision, methodology. **Jianji Li:** conceptualization, supervision, writing – review and editing. **Hongjun Liu** and **Shasha Tan** contributed to the work equally and should be regarded as co‐first authors..

## Funding

This work was supported by the Sichuan Provincial Research Center for the Development of Primary Healthcare Services (Grant No. SWFZ24‐Y‐68).

## Ethics Statement

These publicly accessible, anonymized databases did not require ethical approval or informed consent for this research, which complied with relevant ethical guidelines.

## Conflicts of Interest

The authors declare no conflicts of interest.

## Supporting Information

Additional supporting information can be found online in the Supporting Information section.

## Supporting information


**Supporting Information 1** Figure S1: Study design and analytical workflow.

**Supporting Information 2** Figure S2: Quality control, batch correction, and global cell‐type annotation of the spatially stratified single‐cell atlas.

**Supporting Information 3** Figure S3: Trajectory inference robustness and state program dynamics underlying “evolutionary acceleration” in EGFR‐amplified GBM.

**Supporting Information 4** Figure S4: Sensitivity analysis of PriorityScore2 across alternative weighting schemes.

**Supporting Information 5** Table S1: Patient and sample information for the spatially stratified IDH‐wildtype GBM cohort (GSE286419).

## Data Availability

The data that support the findings of this study are available in Gene Expression Omnibus (GEO) at https://www.ncbi.nlm.nih.gov/geo/query/acc.cgi?acc=GSE286419, accession number GSE286419. These data were derived from publicly available resources: Pai B et al., Cancer Research, 2025.
