# Supplementary figures and images for: Periostin Safeguards EGFR‐Driven Genomic Instability and Sustains the Immune‐Suppressive Niche in Glioblastoma
Source: Hum Mutat. 2026 May 3;2026:9501906. doi: 10.1155/humu/9501906 (PMC13136588; doi:10.1155/humu/9501906)

## Slide 1
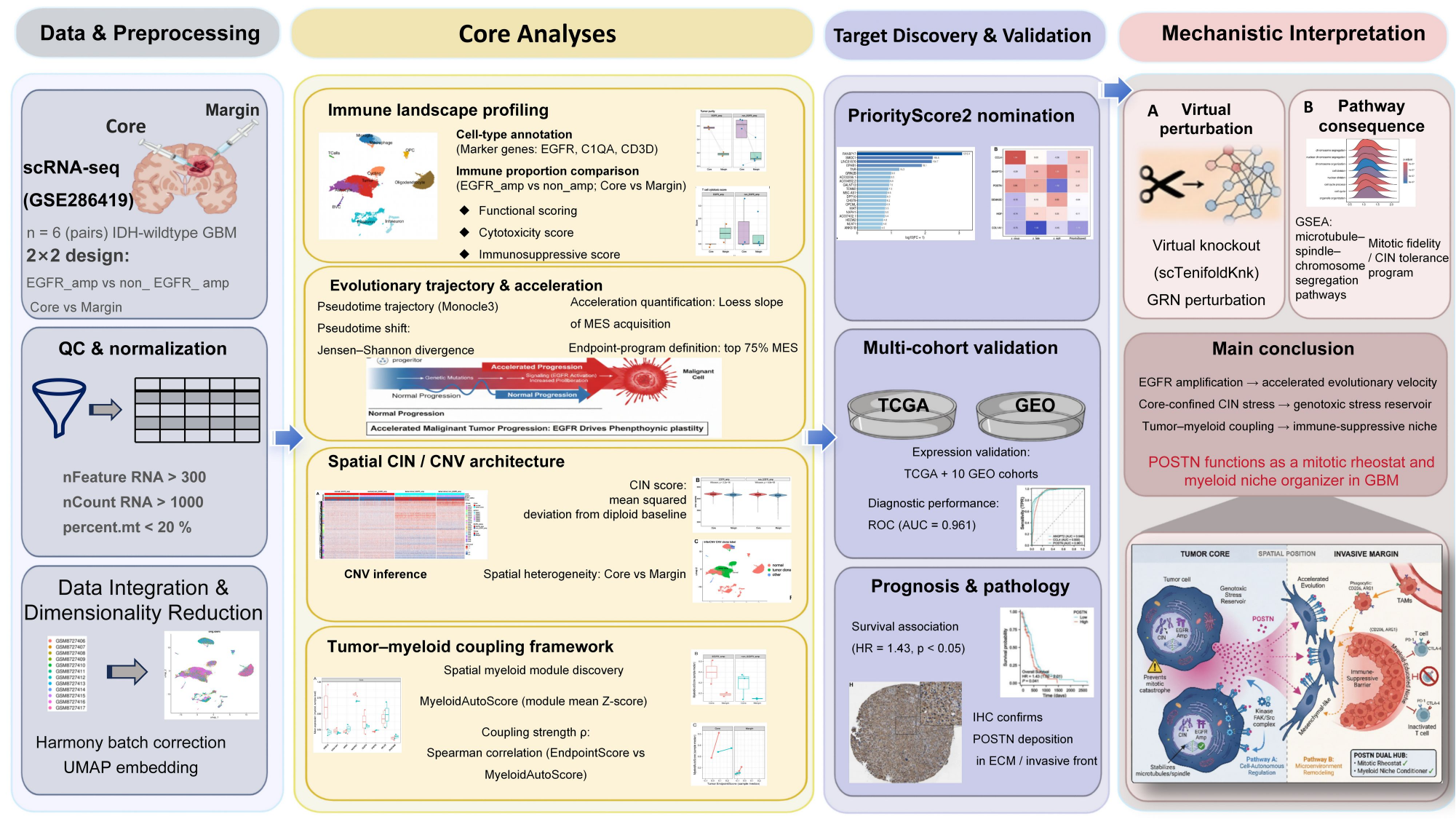

Supplement: Supplementary file 1 — Supporting Information 1 Figure S1: Study design and analytical workflow. [file HUMU-2026-9501906-s004.pptx]

## Slide 1
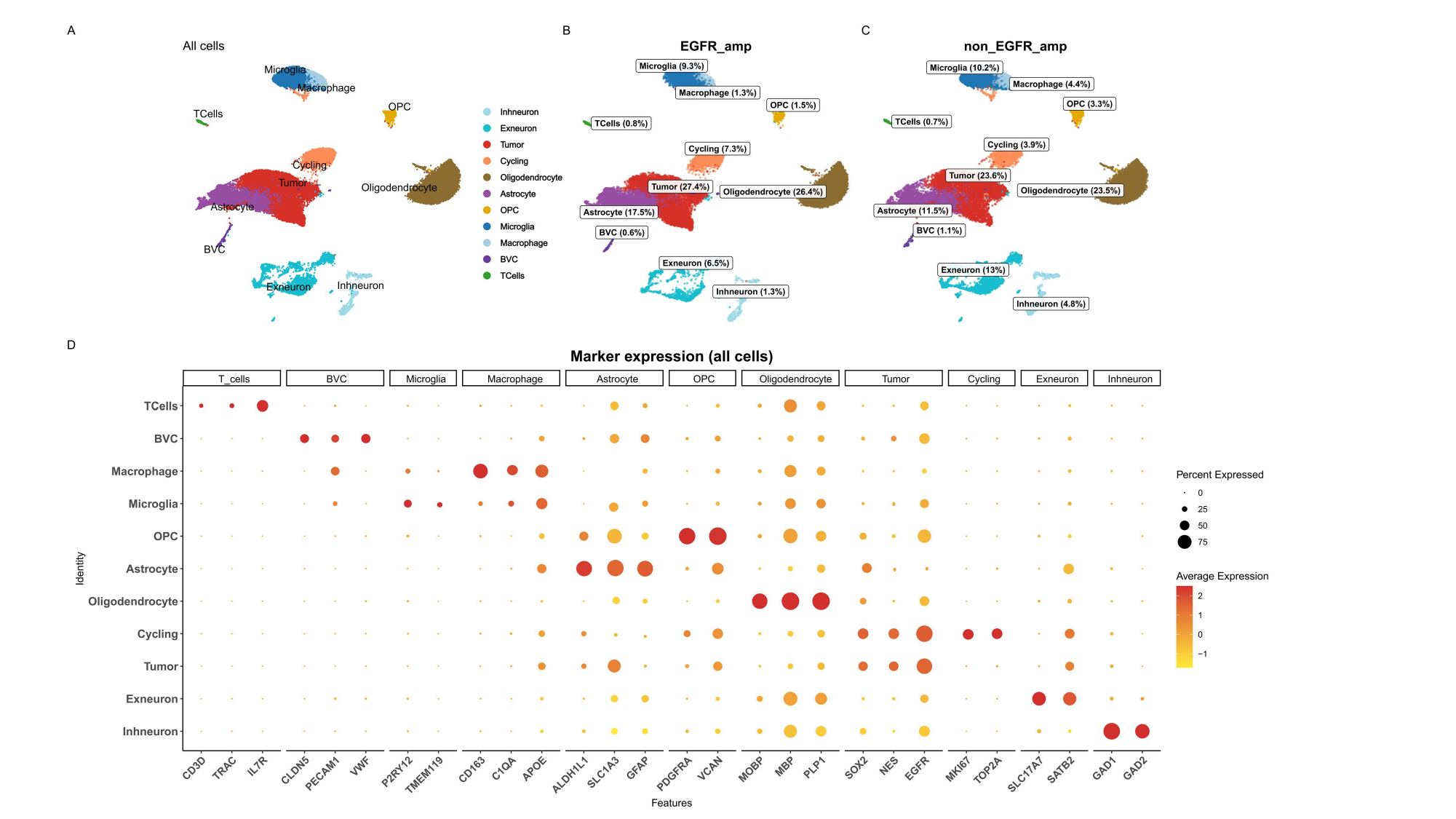

Supplement: Supplementary file 2 — Supporting Information 2 Figure S2: Quality control, batch correction, and global cell‐type annotation of the spatially stratified single‐cell atlas. [file HUMU-2026-9501906-s003.pptx]

## Slide 1
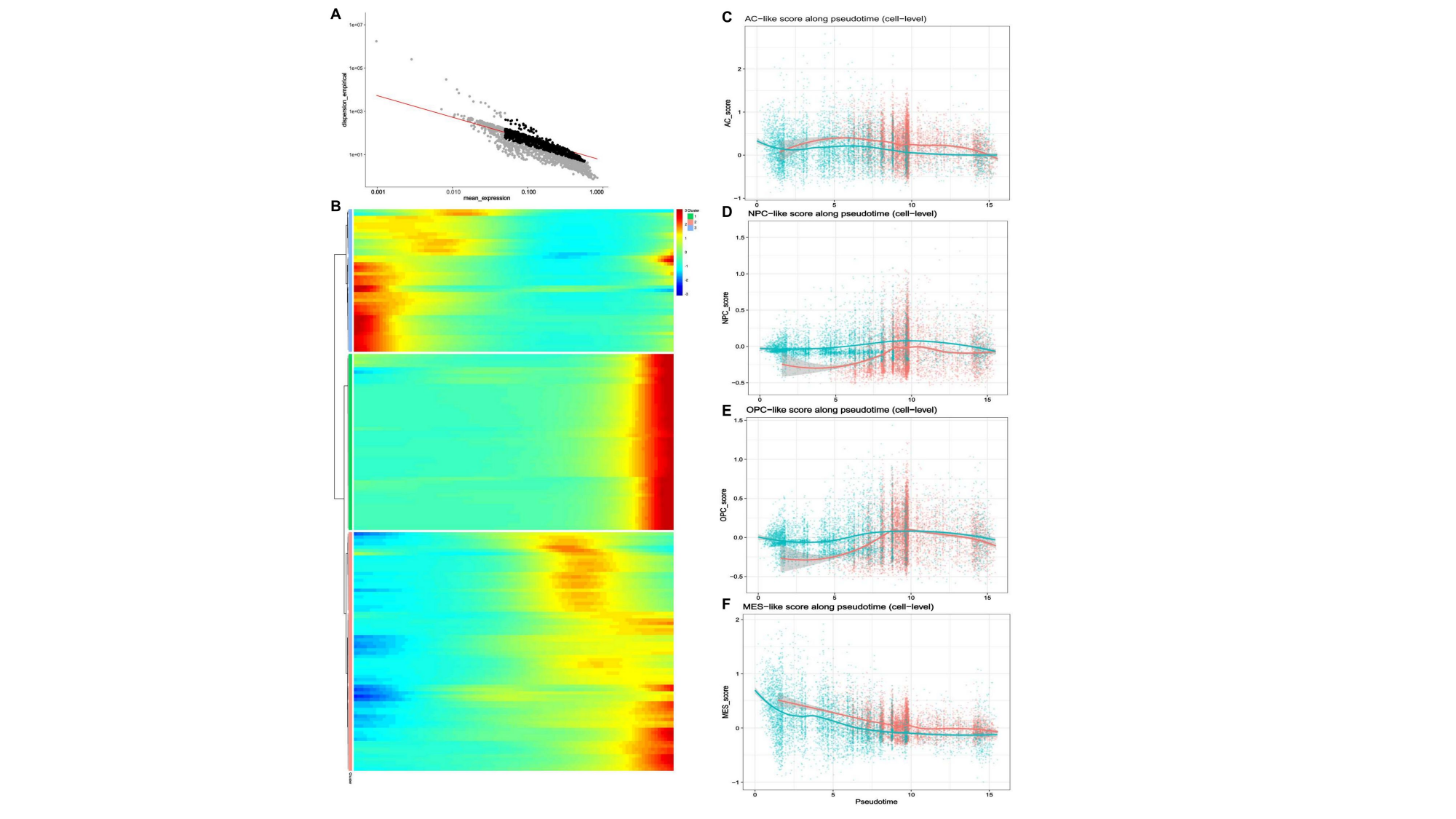

Supplement: Supplementary file 3 — Supporting Information 3 Figure S3: Trajectory inference robustness and state program dynamics underlying “evolutionary acceleration” in EGFR‐amplified GBM. [file HUMU-2026-9501906-s002.pptx]

## Slide 1
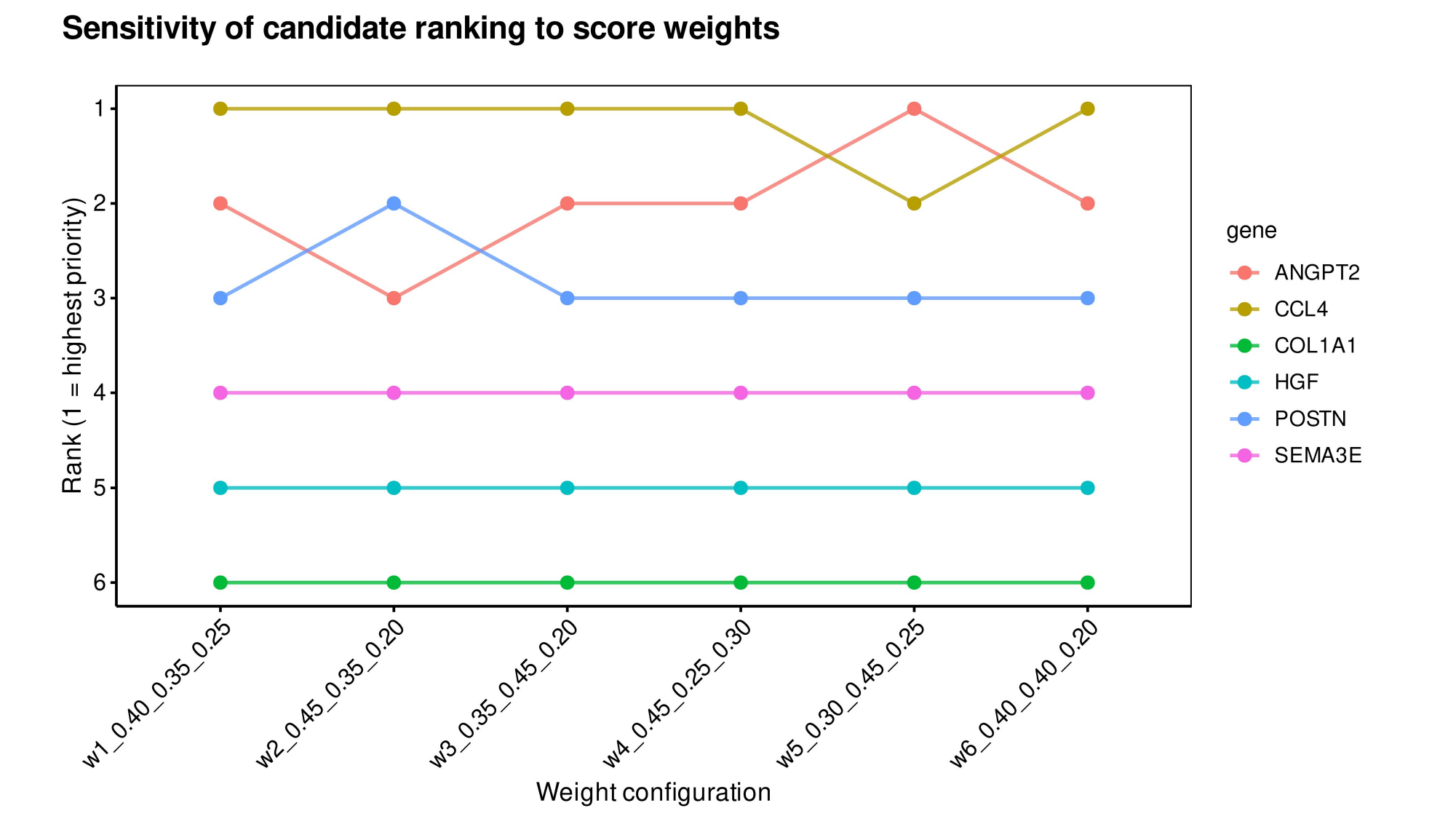

Supplement: Supplementary file 4 — Supporting Information 4 Figure S4: Sensitivity analysis of PriorityScore2 across alternative weighting schemes. [file HUMU-2026-9501906-s001.pptx]
